# Supplementary material for: Content-rich biological network constructed by mining PubMed abstracts
Source: BMC Bioinformatics. 2004 Oct 8;5:147. doi: 10.1186/1471-2105-5-147 (PMC528731; doi:10.1186/1471-2105-5-147)
Supplement: Additional File 2 — The original results of the above study (non-essential files are deleted to keep the file size under the limit set by BMC bioinformatics). [file 1471-2105-5-147-S2.bz2 › chilibotAdditionalFile2/dip20/620ID8598903E11005/html/F7_F3.html]

 


 **F7** and **F3** 
  
Found 158 abstracts in PubMed, retrieved 20.  
 

 What does Google say? 
 PDF only 
| .edu only 

---

**Interactive relationship** (e.g. stimulation, inhibition, etc)

**Neutral relationship**- ...  **F3**  and  **F7**  were able to bind to all Ole e 1 variants.  Ref: 12213332 Mol Immunol, 2002
- Tissue factor  [ **F3** ]  TF is the cellular receptor and cofactor for blood coagulation factor VII  [ **F7** ]  FVII.  Ref: 12823950 Trends Pharmacol Sci, 2003
- BACKGROUND Initiation of the coagulation serine protease cascade in mammalian cells is mediated by tissue factor  [ **F3** ]  TF, which is a cell surface receptor and cofactor for coagulation factor VII  [ **F7** ]  FVII and its activated form FVII FVIIa.  Ref: 11876468 Mol Biotechnol, 2002
- Tissue factor  [ **F3** ]  TF is the main initiator of the coagulation system and functions as a cell surface receptor for coagulation factor VII  [ **F7** ]  FVII.  Ref: 11841337 Biochemistry Mosc , 2002
- One candidate protein to cause coronary NR is tissue factor  [ **F3** ]  TF, which is abundant in atherosclerotic plaque and a cofactor for activated plasma coagulation factor VII  [ **F7** ] .  Ref: 11929768 Blood, 2002

**Non-interactive relationship** (e.g. studied together, co-existance, homology, etc.)

- Although the binding of human IgE to Ole e 1 was not affected by heat treatment, it was shown to be strongly dependent on the integrity of the disulfide bridges and was partially inhibited by  **F3**   **F7**  IgG antibodies, their individual values ranging from 12 to 31% and reaching 53% with their mixture.  Ref: 12213332 Mol Immunol, 2002
- Tissue factor  [ **F3** ]  TF, a transmembrane receptor for the serine protease coagulation factor VII  [ **F7** ]  a FVIIa, is the main initiator of the coagulation cascade.  Ref: 11973337 J Biol Chem, 2002
- A frequent human coagulation Factor VII  [ **F7** ]  mutation A294V, c152 in loop 140s affects the interaction with activators, tissue factor  [ **F3** ]  and substrates.  Ref: 11931672 Biochem J, 2002
- Log transformed values of two qEEG bands, i.e. 2 6 and 6.5 12 Hz, were averaged between adjacent channels frontal  **F3**  and  **F7** , F4 and F8.  Ref: 12207147 Neuropsychobiology, 2002
- Tissue factor  [ **F3** ]  TF, a cell surface associated cofactor and activator of coagulation factor VII  [ **F7** ] , has been implicated in the local and systemic activation of coagulation associated with sepsis.  Ref: 12658585 Vet Clin Pathol< MedlineTA>Vet Clin Pathol, 2002
- Increased SCD was observed at left frontotemporal FT3, bilateral temporoparietal TP3 and TP4 and prefrontal locations F8 F4 and  **F7**   **F3** , suggesting novelty P3 generators located in the left auditory cortex, and bilaterally in temporoparietal and prefrontal association regions.  Ref: 12706218 Brain Res Cogn Brain Res, 2003
- MRCPs were recorded in 20 right handed healthy young subjects with electrodes placed at Fp1, Fp2,  **F7** ,  **F3** , F4, F8, T7, C5, C3, C1, C2, C4, C6, T8, P7, P3, P4, P8, O1 and O2.  Ref: 11856630 Clin Neurophysiol, 2002
- We found that only fractions  **F3**  and  **F7**  inhibit the tested enzyme.  Ref: 12490243 Fitoterapia, 2002
- In such women the arterial intima can express tissue factor  [ **F3** ] , and changes in coagulation factor VII  [ **F7** ]  factor VII and tissue factor  [ **F3** ]  pathway inhibitor TFPI may be deleterious.  Ref: 12456630 Hum Reprod, 2002
- Effects of different progestin regimens in hormone replacement therapy on blood coagulation factor VII  [ **F7** ]  and tissue factor  [ **F3** ]  pathway inhibitor.  Ref: 12456630 Hum Reprod, 2002
- After varimax rotation, we obtained seven independent factors F1 Disinhibition instability, F2 Paranoia hostility,  **F3**  Deficit, F4 Grandiosity psychosis, F5 Elation euphoria, F6 Depression, and  **F7**  Hyper sexuality.  Ref: 12507733 J Affect Disord, 2003
- Among these, tissue factor  [ **F3** ]  and its ligand coagulation factor VII  [ **F7** ] , thrombin and its protease activated receptors, and type II trans membrane serine proteases seem to play a crucial role.  Ref: 12067200 Clin Exp Metastasis, 2002
